# Supplementary figures and images for: An experimental evaluation of the effect of escape gaps on the quantity, diversity, and size of fish caught in traps in Montserrat
Source: PLoS One. 2021 Dec 10;16(12):e0261119. doi: 10.1371/journal.pone.0261119 (PMC8664196; doi:10.1371/journal.pone.0261119)

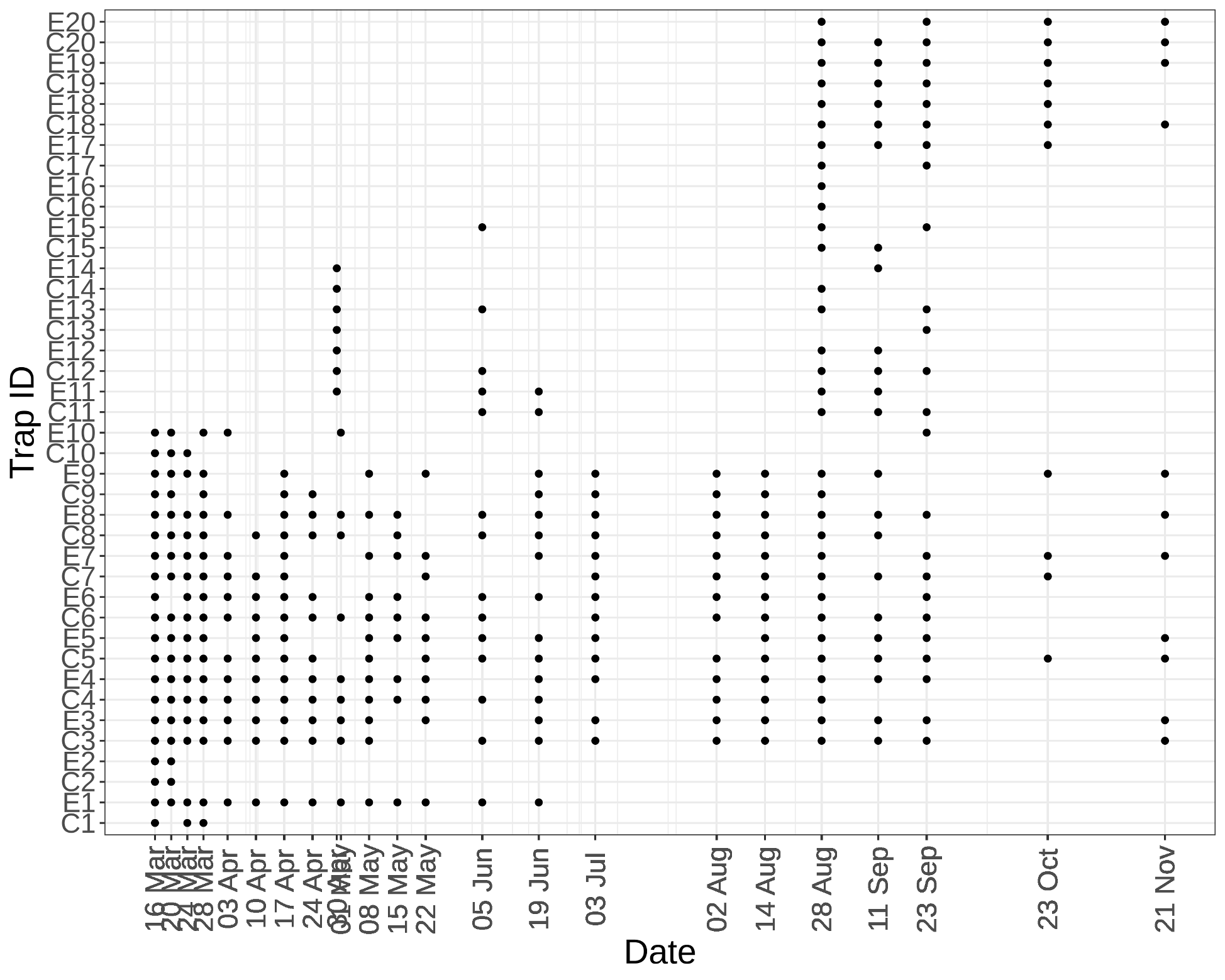

Supplement: S1 Fig — Presence of a dot on a date indicates that the trap was hauled, absence indicates it was not hauled. Experimental (E) and Control (C) traps were deployed in pairs which have the same number, e.g. E1 and C1 are an experimental-control trap pair. (TIF) [file pone.0261119.s001.tif]

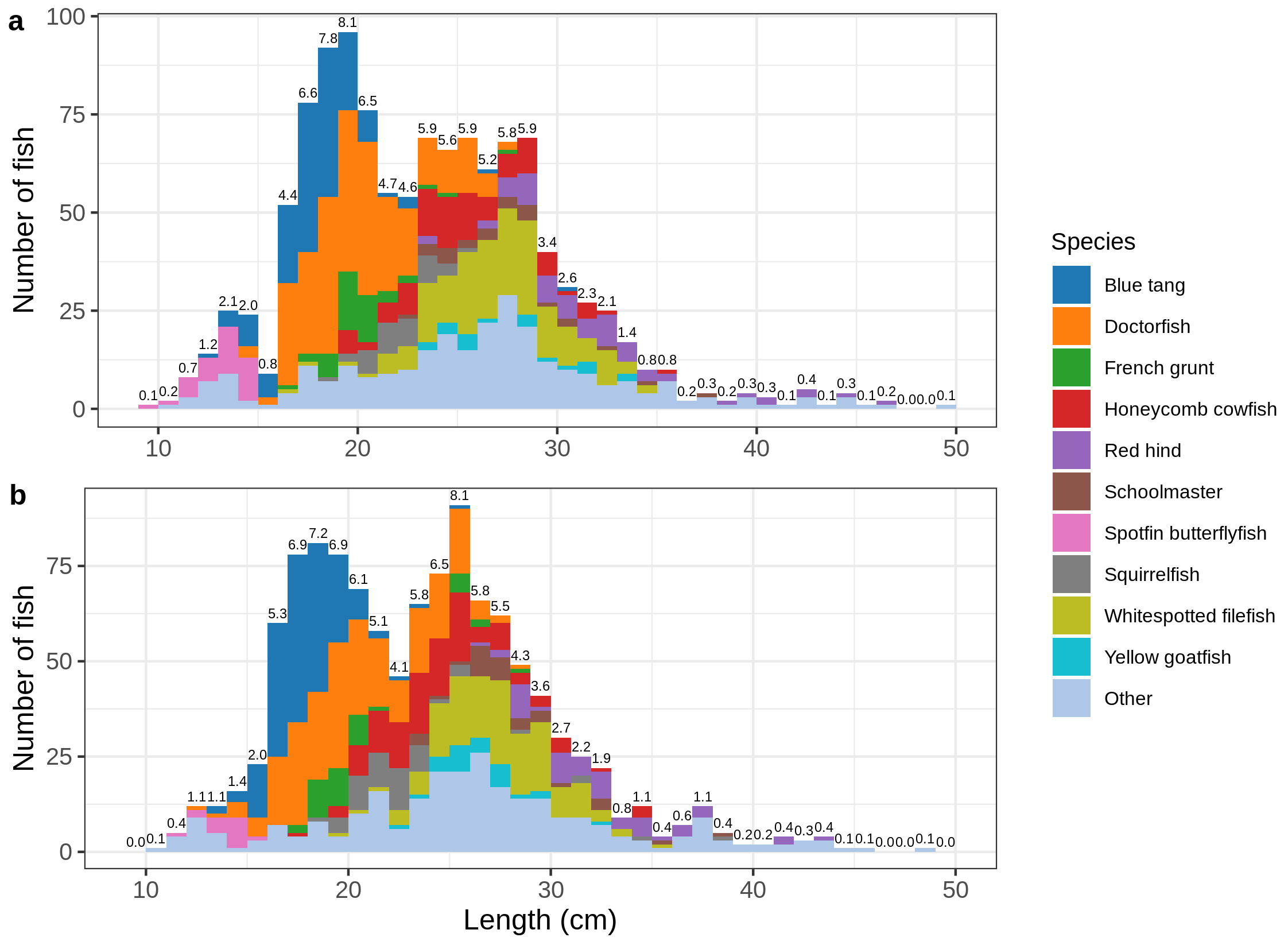

Supplement: S2 Fig — Numbers above bars show percentage of total number of fish that each bar represents. Bar fill colour shows species that were more than 2% of the total number of fish (control and experimental traps catch combined), with all other species classified as “other”. Fish greater than 50 cm (n = 6) have been omitted for clearer visualization. (TIF) [file pone.0261119.s002.tif]

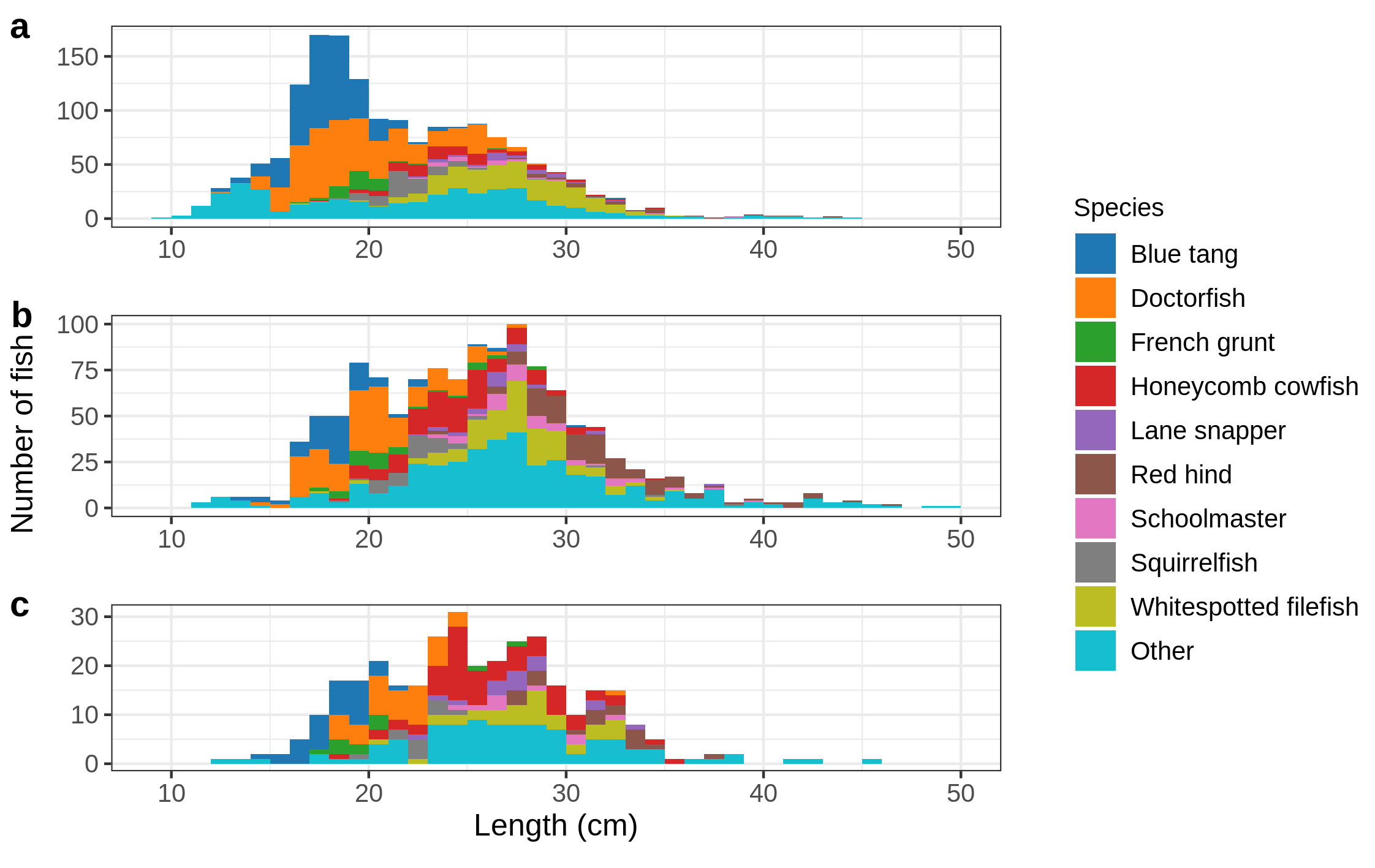

Supplement: S3 Fig — Length-frequency distributions of fish species caught in all trap hauls (excluding zero hauls) with: (a) short soak times (4, 6 and 7 days, n = 148); (b) medium soak times (12 and 14 days, n = 124); (c) long soak times (29 and 30 days, number of hauls = 35). Bar fill colour shows species that were more than 2% of the total number of fish (control and experimental traps catch combined), with all other species classified as “other. Fish larger than 50 cm (n = 7) omitted for clearer visualization. Note different y-axes scales due to different sample sizes. (TIF) [file pone.0261119.s003.tif]
