# Supplementary material for: An experimental evaluation of the effect of escape gaps on the quantity, diversity, and size of fish caught in traps in Montserrat
Source: PLoS One. 2021 Dec 10;16(12):e0261119. doi: 10.1371/journal.pone.0261119 (PMC8664196; doi:10.1371/journal.pone.0261119)
Supplement: S1 Table — Summary of environmental and catch data for control traps (without escape gaps) and experimental traps (with escape gaps). Data for all traps and only trap pairs hauled together are presented. All means are presented ± standard deviation. (DOC) [file pone.0261119.s004.doc]

#### **S1 Table. Extended version of Table 1.** Summary of environmental and catch data for control traps (without escape gaps) and experimental traps (with escape gaps). Data for all traps and only trap pairs hauled together are presented. All means are presented ± standard deviation.

|  | **Only data from paired trap hauls** | | **All traps** | |
| --- | --- | --- | --- | --- |
|  | **Control** | **Experimental** | **Control** | **Experimental** |
| No. of hauls | 135 | 135 | 157 | 179 |
| No. of hauls with zero fish | 11 | 16 | 12 | 17 |
| No. individual fish measurements | 1320 | 1253 | 1503 | 1707 |
| Total no. species | 52 | 43 | 52 | 49 |
| Mean water depth of traps (m) | 36.8 ± 12.9 | 32.5 ± 10.4 | 36.8 ± 12.9 | 32.5 ± 10.4 |
| Mean soak time (days) | 11.0 ± 7.3 | 11.0 ± 7.3 | 11.2 ± 7.4 | 11.3 ± 7.4 |
| Mean trap soak time (days; hauls with zero fish excluded) | 11.9 ± 7.6 | 11.9 ± 7.6 | 11.6 ± 7.5 | 11.6 ± 7.6 |
| Mean number of fish per haul | 9.8 ± 9.6 | 9.3 ± 10.2 | 9.6 ± 9.2 | 9.5 ± 10.0 |
| Mean number of fish per haul ( hauls with zero fish excluded) | 10.8 ± 9.1 | 10.4 ± 9.0 | 10.4 ± 9.2 | 10.5 ± 10.0 |
| Mean length of fish (cm; grouped at individual fish level, hauls with zero fish excluded) | 24.0 ± 7.1 | 24.0 ± 6.1 | 23.7 ± 7.1 | 24.1 ± 5.8 |
| Mean length of fish per haul (cm; grouped at the haul level) | 23.2 ± 8.3 | 22.3 ± 9.3 | 23.3 ± 8.0 | 22.8 ± 8.5 |
| Mean length of fish per haul (cm; grouped at the haul level, hauls with zero fish excluded) | 25.2 ± 4.7 | 25.4 ± 4.7 | 25.2 ± 4.6 | 25.2 ± 4.3 |
| Mean biomass of fish (g; grouped at individual fish level, hauls with zero fish excluded) | 366.5 ± 406.8 | 360.0 ± 302.2 | 355.2 ± 392.2 | 350.0 ± 281.9 |
| Mean total biomass of fish (kg; grouped at the haul level) | 3.4 ± 3.6 | 3.2 ± 3.5 | 3.4 ± 3.5 | 3.3 ± 3.4 |
| Mean total biomass of fish (kg; grouped at the haul level, hauls with zero fish excluded) | 4.0 ± 3.7 | 3.7 ± 3.4 | 3.7 ± 3.5 | 3.7 ± 3.3 |
| Mean number of species per haul | 3.5 ± 2.4 | 3.4 ± 2.6 | 3.6 ± 2.4 | 3.5 ± 2.6 |
| Mean number of species per haul (hauls with zero species excluded) | 4.0 ± 2.3 | 4.0 ± 2.4 | 3.9 ± 2.3 | 3.9 ± 2.5 |
