# Supplementary material for: An experimental evaluation of the effect of escape gaps on the quantity, diversity, and size of fish caught in traps in Montserrat
Source: PLoS One. 2021 Dec 10;16(12):e0261119. doi: 10.1371/journal.pone.0261119 (PMC8664196; doi:10.1371/journal.pone.0261119)
Supplement: S2 Table — All tests were run on data from paired trap hauls only, with and without zero fish hauls included. (DOC) [file pone.0261119.s005.doc]

#### **S2 Table. Statistics results for differences between catch (number, mean length, biomass, number of species, and Shannon diversity index) of fish in control and experimental traps.** All tests were run on data from paired trap hauls only, with and without zero fish hauls included.

| **Variable** | **Data** | **Transformation** | **Test** | **t-statistic for t-test, or V- or W-statistic for Wilcoxon signed rank test (paired and non-paired respectively)** | **df** | **p value** |
| --- | --- | --- | --- | --- | --- | --- |
| Number of fish per haul | Paired hauls | log(x+1) | t-test (paired) | 0.92446 | 134 | 0.3569 |
| Number of fish per haul | Paired hauls | None | Wilcoxon signed rank (paired) | 3925.5 |  | 0.6572 |
| Number of fish per haul | Paired hauls, no zeros | log | t-test (paired) | 0.60669 | 108 | 0.5453 |
| Number of fish per haul | Paired hauls, no zeros | None | Wilcoxon signed rank (paired) | 2412.5 |  | 0.8983 |
| Mean length of fish at individual fish level | Paired hauls | log+1 | t-test (non-paired) | 0.54537 | 2541.3 | 0.5855 |
| Mean length of fish at individual fish level | Paired hauls | None | Wilcoxon signed rank (non-paired) | 842567 |  | 0.9187 |
| Mean length of fish at individual fish level | Paired hauls, no zeros | log | t-test (non-paired) | -0.62196 | 2312.5 | 0.534 |
| Mean length of fish at individual fish level | Paired hauls, no zeros | None | Wilcoxon signed rank (non-paired) | 663154 |  | 0.6882 |
| Mean length of fish at haul level | Paired hauls | log+1 | t-test (paired) | 0.99421 | 134 | 0.3219 |
| Mean length of fish at haul level | Paired hauls | None | Wilcoxon signed rank (paired) | 4776.5 |  | 0.5735 |
| Mean length of fish at haul level | Paired hauls, no zeros | log | t-test (paired) | -0.34335 | 108 | 0.732 |
| Mean length of fish at haul level | Paired hauls, no zeros | None | Wilcoxon signed rank (paired) | 2930.5 |  | 0.8407 |
| Mean weight of fish at individual fish level | Paired hauls | log+1 | t-test (non-paired) | -0.43037 | 2581.2 | 0.667 |
| Mean weight of fish at individual fish level | Paired hauls | None | Wilcoxon signed rank (non-paired) | 817216 |  | 0.1536 |
| Mean weight of fish at individual fish level | Paired hauls, no zeros | log | t-test (non-paired) | -1.2357 | 2309 | 0.2167 |
| Mean weight of fish at individual fish level | Paired hauls, no zeros | None | Wilcoxon signed rank (non-paired) | 646870 |  | 0.1573 |
| Mean total biomass of fish at haul level | Paired hauls | log+1 | t-test (paired) | 0.80255 | 134 | 0.4237 |
| Mean total biomass of fish at haul level | Paired hauls | None | Wilcoxon signed rank (paired) | 4734.5 |  | 0.6386 |
| Mean total biomass of fish at haul level | Paired hauls, no zeros | log | t-test (paired) | -0.58531 | 108 | 0.5596 |
| Mean total biomass of fish at haul level | Paired hauls, no zeros | None | Wilcoxon signed rank (paired) | 3043 |  | 0.8918 |
| Number species per haul | Paired hauls | log+1 | t-test (paired) | 0.78555 | 134 | 0.4335 |
| Number species per haul | Paired hauls | None | Wilcoxon signed rank (paired) | 3336 |  | 0.7403 |
| Number species per haul | Paired hauls, no zeros | log | t-test (paired) | 0.13628 | 108 | 0.8919 |
| Number species per haul | Paired hauls, no zeros | None | Wilcoxon signed rank (paired) | 1925.5 |  | 0.8935 |
| Shannon diversity index | Paired hauls | None | t-test (paired) | 0.64397 | 134 | 0.5207 |
| Shannon diversity index | Paired hauls | None | Wilcoxon signed rank (paired) | 3678 |  | 0.5388 |
| Shannon diversity index | Paired hauls, no zeros | None | t-test (paired) | 0.23149 | 108 | 0.8174 |
| Shannon diversity index | Paired hauls, no zeros | None | Wilcoxon signed rank (paired) | 2660 |  | 0.9123 |
