# Supplementary material for: An experimental evaluation of the effect of escape gaps on the quantity, diversity, and size of fish caught in traps in Montserrat
Source: PLoS One. 2021 Dec 10;16(12):e0261119. doi: 10.1371/journal.pone.0261119 (PMC8664196; doi:10.1371/journal.pone.0261119)
Supplement: S3 Table — Total number of fish and the percentage of the total number of fish in control or experimental traps, and chi-squared test results are shown. (DOC) [file pone.0261119.s006.doc]

**S3 Table. Species with significantly higher total counts in either control or experimental trap (p<0.05 in chi-squared test of equal proportions).** Total number of fish and the percentage of the total number of fish in control or experimental traps, and chi-squared test results are shown.

| **Species name** | **Control trap** | | **Experimental trap** | | **χ^2^** | **df** | **p-value** |
| --- | --- | --- | --- | --- | --- | --- | --- |
|  | **No. of fish** | **% of total** | **No. of fish** | **% of total** |  |  |  |
| **More fish in experimental trap** | | | | | | | |
| Spanish hogfish (*Bodianus rufus*) | 0 | 0.0 | 11 | 1.0 | 11.5 | 1 | 0.001 |
| Coney (*Cephalopholis fulva*) | 2 | 0.2 | 8 | 0.7 | 3.9 | 1 | 0.049 |
| Princess parrotfish (*Scarus taeniopterus*) | 5 | 0.4 | 20 | 1.8 | 9.8 | 1 | 0.002 |
| Yellowtail snapper (*Ocyurus chrysurus*) | 10 | 0.8 | 22 | 1.9 | 5.1 | 1 | 0.024 |
| **More fish in control trap** | | | | | | | |
| Queen angelfish (*Holacanthus ciliaris*) | 11 | 0.9 | 3 | 0.3 | 4.3 | 1 | 0.039 |
| Almaco jack (*Seriola rivoliana*) | 12 | 1.0 | 1 | 0.1 | 8.9 | 1 | 0.003 |
| Horse-Eye jack (*Caranx latus*) | 13 | 1.1 | 0 | 0.0 | 12.5 | 1 | 0.000 |
| Black durgon (*Melichthys niger*) | 25 | 2.1 | 0 | 0.0 | 24.2 | 1 | 0.000 |
| Spotfin butterflyfish (*Chaetodon ocellatus*) | 36 | 3.0 | 16 | 1.4 | 7.0 | 1 | 0.008 |
