# Supplementary material for: An experimental evaluation of the effect of escape gaps on the quantity, diversity, and size of fish caught in traps in Montserrat
Source: PLoS One. 2021 Dec 10;16(12):e0261119. doi: 10.1371/journal.pone.0261119 (PMC8664196; doi:10.1371/journal.pone.0261119)
Supplement: S4 Table — Data are from paired trap hauls. (DOC) [file pone.0261119.s007.doc]

**S4 Table. Results of Kolmogorov-Smirnov tests comparing length-frequency distributions of species in control and experimental traps.** Data are from paired trap hauls.

| **Species name** | **D_max_** | **p-value** |
| --- | --- | --- |
| Banded butterflyfish (*Chaetodon striatus*) | 0.18 | 0.92 |
| Black margate (*Anisotremus surinamensis*) | 0.28 | 0.40 |
| Blue tang (*Acanthurus coeruleus*) | 0.06 | 0.95 |
| Bluestriped grunt (*Haemulon sciurus*) | 0.29 | 0.59 |
| Doctorfish (*Acanthurus chirurgus*) | 0.09 | 0.27 |
| French grunt (*Haemulon flavolineatum*) | 0.18 | 0.50 |
| Honeycomb cowfish (*Acanthostracion polygonius*) | 0.14 | 0.28 |
| Lane snapper (*Lutjanus synagris*) | 0.31 | 0.40 |
| Pluma porgy (*Calamus pennatula*) | 0.46 | 0.10 |
| Queen triggerfish (*Balistes vetula*) | 0.27 | 0.67 |
| Red hind (*Epinephelus guttatus*) | 0.14 | 0.66 |
| Rock beauty (*Holacanthus tricolor*) | 0.49 | 0.07 |
| Schoolmaster (*Lutjanus apodus*) | 0.22 | 0.48 |
| Smooth trunkfish (*Lactophrys triqueter*) | 0.27 | 0.77 |
| Spotfin butterflyfish (*Chaetodon ocellatus*) | 0.26 | 0.46 |
| Squirrelfish (*Holocentrus adscensionis*) | 0.13 | 0.87 |
| Whitespotted filefish (*Cantherhines macrocerus*) | 0.08 | 0.68 |
| Yellow goatfish (*Mulloidichthys martinicus*) | 0.35 | 0.12 |
