# Supplementary material for: An experimental evaluation of the effect of escape gaps on the quantity, diversity, and size of fish caught in traps in Montserrat
Source: PLoS One. 2021 Dec 10;16(12):e0261119. doi: 10.1371/journal.pone.0261119 (PMC8664196; doi:10.1371/journal.pone.0261119)
Supplement: S5 Table — Only species for which reliable, local, length at maturity (Lmat; length at which at least 50% of individuals are mature) data are available are included. Data are from paired trap hauls. Statistic results (V-statistic and p-value) are from paired Wilcoxon signed rank tests of count data. (DOC) [file pone.0261119.s008.doc]

**S5 Table. Percent of fish caught in control and experimental traps less than length at maturity.** Only species for which reliable, local, length at maturity (L_mat_; length at which at least 50% of individuals are mature) data are available are included. Data are from paired trap hauls. Statistic results (V-statistic and p-value) are from paired Wilcoxon signed rank tests of count data.

| **Species name** | **Percent of fish species less than L_mat_ in control trap** | **Percent of fish species less than L_mat_ in experimental trap** | **More immature fish in control trap?** | **V-statistic** | **p-value** |
| --- | --- | --- | --- | --- | --- |
| Blue tang (*Acanthurus coeruleus*) | 2.7 | 0.0 | True | 6 | 0.18 |
| Doctorfish (*Acanthurus chirurgus*) | 67.3 | 60.9 | True | 793.5 | 0.34 |
| French grunt (*Haemulon flavolineatum*) | 4.5 | 2.6 | True | 4 | 0.79 |
| Red hind (*Epinephelus guttatus*) | 39.3 | 31.4 | True | 193.5 | 0.65 |
| Squirrelfish (*Holocentrus adscensionis*) | 100.0 | 92.0 | True | 95.5 | 1 |
| Queen triggerfish (*Balistes vetula*) | 15.0 | 18.2 | False | 5 | 1 |
| Bluestriped grunt (*Haemulon sciurus*) | 0.0 | 0.0 | Equal | NA | NA |
| Lane snapper (*Lutjanus synagris*) | 0.0 | 0.0 | Equal | NA | NA |
| Schoolmaster (*Lutjanus apodus*) | 0.0 | 0.0 | Equal | NA | NA |
