# Supplementary material for: An experimental evaluation of the effect of escape gaps on the quantity, diversity, and size of fish caught in traps in Montserrat
Source: PLoS One. 2021 Dec 10;16(12):e0261119. doi: 10.1371/journal.pone.0261119 (PMC8664196; doi:10.1371/journal.pone.0261119)
Supplement: S6 Table — Random effects in all models were date and individual trap ID.—p > 0.05; * p < 0.05; ** p < 0.01; *** p < 0.001. (DOC) [file pone.0261119.s009.doc]

#### **S6 Table. Significance of fixed effects considered in the global mixed effect models.** Random effects in all models were date and individual trap ID. - p > 0.05; * p < 0.05; ** p < 0.01; *** p < 0.001.

| **Fixed effect** | **Mean Catch Length** | **Total Catch Biomass (log+1)** | **No. of fish in catch** | **Species richness** |
| --- | --- | --- | --- | --- |
| (log) Soak time | *** | * | - | - |
| Trap design | - | - | - | - |
| Location | * | - | - | - |
| Escape gap | - | - | - | - |
