# Supplementary material for: An experimental evaluation of the effect of escape gaps on the quantity, diversity, and size of fish caught in traps in Montserrat
Source: PLoS One. 2021 Dec 10;16(12):e0261119. doi: 10.1371/journal.pone.0261119 (PMC8664196; doi:10.1371/journal.pone.0261119)
Supplement: S7 Table — Random effects in all models were date and individual trap ID. (DOC) [file pone.0261119.s010.doc]

**S7 Table. Linear mixed effects model summaries for global models.** Random effects in all models were date and individual trap ID.

|  | **Mean catch length (cm)** | | | | **log(total catch biomass (kg))** | | | | **Number of fish** | | | | **Number of species** | | | |
| --- | --- | --- | --- | --- | --- | --- | --- | --- | --- | --- | --- | --- | --- | --- | --- | --- |
| *Predictors* | *Estimates* | *CI* | *Statistic* | *p* | *Estimates* | *CI* | *Statistic* | *p* | *Incidence*  *Rate*  *Ratios* | *CI* | *Statistic* | *p* | *Incidence*  *Rate*  *Ratios* | *CI* | *Statistic* | *p* |
| Intercept | 17.34 | 14.21 – 20.46 | 10.87 | **<0.001** | 0.54 | 0.02 – 1.06 | 2.05 | **0.040** | 8.25 | 4.12 – 16.50 | 5.96 | **<0.001** | 3.24 | 2.13 – 4.94 | 5.48 | **<0.001** |
| Design (V) | 1.57 | -0.19 – 3.32 | 1.75 | 0.080 | 0.06 | -0.23 – 0.36 | 0.43 | 0.670 | 0.91 | 0.61 – 1.37 | -0.45 | 0.654 | 0.89 | 0.67 – 1.16 | -0.87 | 0.384 |
| log(Soak time) | 2.80 | 1.81 – 3.79 | 5.53 | **<0.001** | 0.32 | 0.15 – 0.50 | 3.71 | **<0.001** | 1.12 | 0.90 – 1.40 | 0.99 | 0.325 | 1.11 | 0.99 – 1.25 | 1.78 | 0.075 |
| Location (windward) | 2.64 | 0.46 – 4.83 | 2.38 | **0.018** | -0.15 | -0.51 – 0.20 | -0.86 | 0.392 | 0.65 | 0.40 – 1.06 | -1.71 | 0.087 | 0.72 | 0.51 – 1.03 | -1.81 | 0.071 |
| Escape gaps (present) | -0.04 | -1.12 – 1.05 | -0.06 | 0.949 | 0.00 | -0.20 – 0.20 | 0.00 | 0.998 | 1.00 | 0.76 – 1.32 | 0.02 | 0.985 | 1.02 | 0.83 – 1.24 | 0.16 | 0.871 |
| N | 37 TrapID | | | | 37 TrapID | | | | 37 TrapID | | | | 37 TrapID | | | |
|  | 23 Date_YMD | | | | 23 Date_YMD | | | | 23 Date_YMD | | | | 23 Date_YMD | | | |
| Observations | 307 | | | | 307 | | | | 30 | | | | 307 | | | |
| Marginal R^2^/ Conditional R^2^ | 0.143 / 0.220 | | | | 0.092 / 0.290 | | | | 0.026 / 0.21 | | | | 0.032 / 0.162 | | | |
| AIC | 1745.331 | | | | 561.611 | | | | 2054.669 | | | | 1341.643 | | | |
