# Supplementary material for: An experimental evaluation of the effect of escape gaps on the quantity, diversity, and size of fish caught in traps in Montserrat
Source: PLoS One. 2021 Dec 10;16(12):e0261119. doi: 10.1371/journal.pone.0261119 (PMC8664196; doi:10.1371/journal.pone.0261119)
Supplement: S8 Table — Random effects in all models were date and individual trap ID. (DOC) [file pone.0261119.s011.doc]

**S8 Table. Linear mixed effects model summaries for most parsimonious models.** Random effects in all models were date and individual trap ID.

|  | **Mean catch length (cm)** | | | | **log(total catch biomass (kg))** | | | | **Number of fish** | | | | **Number of species** | | | |
| --- | --- | --- | --- | --- | --- | --- | --- | --- | --- | --- | --- | --- | --- | --- | --- | --- |
| *Predictors* | *Estimates* | *CI* | *Statistic* | *p* | *Estimates* | *CI* | *Statistic* | *p* | *Incidence*  *Rate*  *Ratios* | *CI* | *Statistic* | *p* | *Incidence*  *Rate*  *Ratios* | *CI* | *Statistic* | *p* |
| Intercept | 19.31 | 16.92 – 21.70 | 15.85 | **<0.001** | 0.58 | 0.17 – 0.98 | 2.80 | **0.005** | 9.88 | 8.29 – 11.77 | 25.61 | **<0.001** | 2.88 | 2.15 – 3.84 | 7.16 | **<0.001** |
| log(Soak time) | 2.58 | 1.57 – 3.59 | 4.99 | **<0.001** | 0.32 | 0.15 – 0.49 | 3.65 | **<0.001** |  |  |  |  | 1.13 | 1.01 – 1.27 | 2.10 | **0.036** |
| Location  (windward) |  |  |  |  |  |  |  |  | 0.70 | 0.47 – 1.04 | -1.75 | 0.080 | 0.79 | 0.59 – 1.05 | -1.60 | 0.111 |
| N | 37 TrapID | | | | 37 TrapID | | | | 37 TrapID | | | | 37 TrapID | | | |
|  | 23 Date_YMD | | | | 23 Date_YMD | | | | 23 Date_YMD | | | | 23 Date_YMD | | | |
| Observations | 307 | | | | 307 | | | | 307 | | | | 307 | | | |
| Conditional R^2^/ Marginal R^2^ | 0.125 / 0.217 | | | | 0.088 / 0.294 | | | | 0.018 / 0.210 | | | | 0.028 / 0.160 | | | |
| AIC | 1744.923 | | | | 557.712 | | | | 2050.106 | | | | 1338.416 | | | |
