# Supplementary material for: An experimental evaluation of the effect of escape gaps on the quantity, diversity, and size of fish caught in traps in Montserrat
Source: PLoS One. 2021 Dec 10;16(12):e0261119. doi: 10.1371/journal.pone.0261119 (PMC8664196; doi:10.1371/journal.pone.0261119)
Supplement: Attachment — Submitted filename: Flower_Response to reviewers.docx [file pone.0261119.s012.docx]

# Response to reviewers (PONE-D-20-22876)

*Reviewer comments in italics*. **Responses in bold.**

**Reviewer #1:**

*While the MS has all the technical issues, seems that its questions has been responded in previous papers. I see only a question which shows an interest (escape gaps vs soak time) but the dedication on this is low and it cann't support an original research paper, but maybe a short communication.*

**Response:**

**The reviewer concluded that the paper was scientifically sound, but essentially lacked novelty, since the effect of escape gaps on catch in fish traps has been investigated before. However PLOSONE’s publication criteria state “We evaluate submitted manuscripts on the basis of methodological rigor and high ethical standards, regardless of perceived novelty”, therefore rejection because of lack of novelty does not seem to be a valid reason, although the reviewer does not provide any other reasoning in their incredibly brief comments. Furthermore, as the reviewer points out, we also cover the impact of soak time of trap catches, which is a novel result. Beyond this, the results contribute to a very limited body of research on escape gaps, which we also summarize in the introduction.**

**Reviewer #2:**

*This paper on the effect of escape gaps on the quantity, diversity, and size of fish caught in traps is an important attempt to contribute to better fishing practices and a more sustainable fishery. However, there several flaws that unless solved does not allow the recommendation for publication.*

*The most important is the experimental design which is not clear.*

**We have revised the manuscript to clarify all the points raised as follow:**

*How many traps were deployed in each site, how many sites, what were the depths, the traps were baited or not (if so with which bait?).*

**Lines 153-154 added text to clarify: “....and each experimental-control trap pair (20 pairs in total) was deployed at a different site (Fig 1).”**

**Lines 158-159 added text: “**Trap depths were recorded using a depth sounder **and were in the range 15.2 to 50.3m”**

**We also include mean trap depths in Table 2. Depth information for individual traps is available in the data which we make freely available.**

**Lines 155-156 added text: “Traps were not baited due to inconsistent availability of the bait that some fishermen use (coconut).”**

*The sites were similar to the ones used by the fishermen, but what about the setting times, the soak time and other specificities?*

**Lines 165-167 already state: “**Soak time was initially set at 4 days, but was increased to 1 week, and finally 2 weeks to more closely match realistic soak times used by local fishers...**”**

**And see added text on lines 155-156 regarding bait.**

*What was the accuracy of the measurements of length and weight?*

**Lines 171-172 added text: “**weight **(to the nearest gram)** and length **(to the nearest 0.5 cm)** of each fish was recorded at the landing site**.”**

*How were the traps set? What was the distance between them?*

**Lines 154 - 155 added text: “with each pair next to each other and individually tethered to a buoy on the surface.”**

*What was the mesh size used?*

**Lines 151 - 152 added text:** “**All traps were made using 1.5 inch (3.8 cm) wire mesh,** and fitted with escape panels to minimize ghost fishing impacts if lost.”

*How many replicates were used?*

**Lines 169-170 added text: “There were a total of 23 days when traps were hauled between 16^th^ March 2016 and 21^st^ November 2018 (S1 Fig).”**

**The supplementary Figure S1 shows exactly which traps were hauled on each day. See added text previously for number of experimental-control trap pairs.**

*Then why the use of fixed thresholds (20 and 15cm) to evaluate the effect on smaller individuals?*

*What about sizes of first maturity? This way we are talking only about small and large fish independently of their state of maturity.*

**As we state in the Methods (lines 194-197) “We also compared the distributions of only small-sized classes of fish (<20 cm length and <15 cm length) as we expected the biggest difference between control and experimental traps to be in the catch of narrower, and therefore shorter, fish.”**

**However, we agree that using sizes at maturity as a reference point is also a useful comparison to evaluate whether the escape gaps improved the sustainability of the trap fishery. We therefore added the following analysis:**

**Lines 201-207 (Methods):**

**“To see if the experimental traps would catch a lower proportion of immature fish, we compared the percentage of fish caught below the length at maturity (L_mat_; length at which at least 50% of individuals are mature) in control and experimental traps hauled as pairs. We used L_mat_ values from two sources that had relatively local, reliable information [33,42], providing L_mat_ values for 9 species, representing 54% of the total catch by number. For these 9 species, we compared the number of fish less than L_mat_ in paired control and experimental traps using paired Wilcoxon-signed rank tests since the data were non-normally distributed.”**

**Lines 307-311 (Results):**

**“Of the nine species for which we had length at maturity values, 5 had a higher percentage of immature fish (more fish less than L_mat_) caught in the control traps than the experimental traps, 1 species had a higher percentage in the experimental traps, and the remaining 3 were all caught above L_mat_ in both trap types, though none of the differences were statistically significant (p > 0.1 in all cases; S5 Table).”**

**S5 Table. Percent of fish caught in control and experimental traps less than length at maturity.** Only species for which reliable, local, length at maturity (L_mat_; length at which at least 50% of individuals are mature) data are available are included. Data are from paired trap hauls. Statistic results (V-statistic and p-value) are from paired Wilcoxon signed rank tests of count data.

| **Species name** | **Percent of fish species less than L_mat_ in control trap** | **Percent of fish species less than L_mat_ in experimental trap** | **More immature fish in control trap?** | **V-statistic** | **p-value** |
| --- | --- | --- | --- | --- | --- |
| Blue tang (*Acanthurus coeruleus*) | 2.7 | 0.0 | True | 6 | 0.18 |
| Doctorfish (*Acanthurus chirurgus*) | 67.3 | 60.9 | True | 793.5 | 0.34 |
| French grunt (*Haemulon flavolineatum*) | 4.5 | 2.6 | True | 4 | 0.79 |
| Red hind (*Epinephelus guttatus*) | 39.3 | 31.4 | True | 193.5 | 0.65 |
| Squirrelfish (*Holocentrus adscensionis*) | 100.0 | 92.0 | True | 95.5 | 1 |
| Queen triggerfish (*Balistes vetula*) | 15.0 | 18.2 | False | 5 | 1 |
| Bluestriped grunt (*Haemulon sciurus*) | 0.0 | 0.0 | Equal | NA | NA |
| Lane snapper (*Lutjanus synagris*) | 0.0 | 0.0 | Equal | NA | NA |
| Schoolmaster (*Lutjanus apodus*) | 0.0 | 0.0 | Equal | NA | NA |

*What is the scientific base for the classification of fish based on thickness? Is there any study on the body shape of the fish that support this classification? According to the results, it is evidenced that this classification did not make much sense for the current objectives.*

**Our original classification was somewhat subjective. We have revised this part of the analysis, using body compression descriptions in a curated Caribbean fish database (**[**https://biogeodb.stri.si.edu/caribbean/en/pages**](https://biogeodb.stri.si.edu/caribbean/en/pages)**) with edits to the body compression classifications as suggested by the database maintainer who is a Caribbean reef fish expert:**

**Lines 214 - 223 (Methods): “To examine the effect of the escape gaps on retention of fish according to body width, we classified each species using descriptions of body shape from a curated database or Caribbean fish [41], with some modifications to the classification after communication with the database maintainer (pers. Comms. R. Robertson, 11^th^ March 2021). Fish were classified from narrowest (most compressed) to widest (not compressed) with those described as “very compressed” or “strongly compressed” classified as “very compressed”, those as "moderately compressed” or “somewhat compressed" classified as "moderately compressed", those as “compressed” classified as “slightly compressed”, and those as "thick” or “robust" as "not compressed". Differences in the proportions of fish in each group caught in control and experimental traps were examined using chi-squared tests.”**

**Lines 318 - 326 (Results): “The proportion of very compressed (narrowest) fish found in control traps was significantly higher than that in experimental traps (χ^2^(1) = 4.3, p = 0.04, Fig 5). Conversely, the proportion of not compressed (widest) fish was significantly higher in experimental traps than control traps (χ^2^(1) = 4.6, p = 0.03). The proportions of fish classified as moderately and slightly compressed were not significantly different between control and experimental traps.”**

**Fig 5. Proportion of fish in control and experimental traps classified by body width. Asterisk above bars indicates there was a significant difference between control and experimental traps (p< 0.05, chi-squared test)**

*If there was no direct involvement from the academy in the experimental fishing - what was done by the responsible researchers to ensure the scientific quality of the work? This is extremely important, and the recommendation for publication would depend very much on the justifications and explanations about this issue.*

**Numerous papers are published (including in PLOSONE) using citizen science data and data collected by non-university affiliated authors** [**(e.g. Roelfsema et al. 2016; Oviedo & Bursztyn 2017; Fulton et al. 2019)**](https://www.zotero.org/google-docs/?rlYFfF)**. Furthermore, all our data collection was done by a single person (co-author Keinan James), eliminating between observer differences in measurement, and he received training before starting the data collection process. We have added the following text (lines 175-176) to clarify this:**

**“A single data collector (K.J.) collected all data and received training before the experiment commenced.”**

*There also several statements in the discussion that are not entirely accurate, such as about comparisons between leeward and windward fish abundance and diversity, and unsupported supposition about the efficiency of the traps in function of soak time.*

**We address these comments in the detailed line comments below.**

*In resume, I think the paper can not be recommended for publication in its current state, but the authors could resubmit after significant changes.*

**We feel we have thoroughly addressed all of the reviewers concerns, and appreciate the detailed comments.**

*Other more specific issues and comments:*

*L49-51 If MPA is hard to enforce, why would the selectivity or the soak time or the fishing area be in a better solution?*

*Most of these solutions involve enforcement as well and, in addition to MPA, involves costs for the fisherman in terms o fishing gear (new or modified) and eventually less economic yield…*

**We have already provided two supporting references for this statement and do not believe the sentence implies that gear-based management does not have enforcement or cost challenges. We have removed the word “often” to acknowledge that the acceptability of such solutions is context dependent.**

**“**Gear-based management, where fishing gear is regulated to control selectivity, or the timing and location of gear deployment is controlled, can **~~often~~** be more acceptable to fishers and lower cost to implement [9,11].**”**

*L57-58 Needs a citation*

**Added citation of: Mahon R, Hunte W. Trap mesh selectivity and the management of reef fishes. Fish Fish. 2001;2: 356–375.**

*L95-L98 The reduction of the percentage of juvenile fish should be one of the hypothesis. The reduction of the mean length is not exactly the same.*

**Agreed. We have added (lines 97 - 98): “5) The numbers of juvenile fish caught in experimental traps would be lower than in control traps”**

*L99-102 Not clear.*

**Previous paragraph:**

“Unlike past studies, our traps were paired but were set in locations with different depths and had differing soak times (time between hauls when the catch is removed) over the length of the experiment, and two trap designs were used, allowing us to also examine the impact of these factors on catch for traps with and without escape gaps.”

**Rewritten (lines 100 - 104):**

**“Our experimental design was similar to previous experiments, with traps deployed in pairs, but we also tested two trap designs, set trap pairs at differing depths, and had variable soak times (time between hauls when the catch is removed) over the length of the experiment. This allowed us to explore the impacts of these factors on catch for traps with and without escape gaps.”**

*L134-135 Needs a citation. Is there any information about the condition of the discarded fish, ie if they are discarded alive in good conditions of survivorship?*

**Line 136 added text: “(pers. obs. L.T., 19 June 2015)”**

**There is very limited information on Montserrat’s fisheries and unfortunately no information about discarded fish condition.**

*L235-236 How come we have different hauls for control and treatment if the traps were deployed as pairs? If one was lost the haul should not count?*

**The table shows data from both only paired trap hauls (same number of hauls for control and experimental traps), and all trap hauls, as indicated in the column headings. The all trap hauls data has different numbers of hauls for control and experimental traps because of lost traps and the fishers being unable to haul traps due to bad weather on some trips, as described in the Methods:**

**“Hauling of traps was sometimes delayed due to bad weather, and individual traps could not always be hauled on a trip due to ocean conditions and problems locating them. ”**

*Table 2 If the traps were deployed together in pairs how come there are different mean depths? Wasn't it supposed to be equal/similar?*

**Lines 159-161 added text (Methods): “Although trap pairs were deployed at the same location, natural movement of the traps due to ocean conditions and the steep shelf slope around Montserrat could result in differing depths within a trap pair.”**

**Traps were deployed in pairs and depths should therefore be similar, however rough seas and natural movement of the traps underwater mean that traps were not necessarily always right next to each other. Furthermore, the shelf slope is quite steep in some areas, therefore a small horizontal distance between traps can translate into a several meter difference in depth. Given this, the difference between the mean depth of experimental and control traps of 4.3m is quite small.**

*Fig. 3 What is the point of having a figure with the total catch? Better to be removed.*

**The figure provides a visual summary of the principal species caught, which is useful in the context of the later length- and body width-based analysis. We are happy to move it to the supplementary materials if that is preferred.**

*L279- …This is a scientific paper and thus all the species should be referred with its scientific name at least the first time they are mentioned.*

**Scientific names added to all species the first time they are mentioned in the paper and in all Tables and Figures.**

*L293 In table 3 only soak time is a significant predictor, not location.*

*What was the value of the experimental effect?*

**Line 328 changed text: “**Of the four fixed effects considered in ~~the~~ **all** models, only soak time and location were significant predictors (Table 3 and S3 Table)**”**

**We are summarizing the results of both the global and most parsimonious models in this sentence. Table 3 only has results for the most parsimonious model, S3 Table (now S6 Table) has the results for the global model. Experimental effect values are given in the next paragraph and Tables S7 and S8.**

*L329-331 The results showed that the pre-classification was not accurate.*

**We have revised our method of classifying fish according to body shape (see detailed response to earlier comment). This new classification appears to have been more suitable, with the results fitting our expectation that fewer of the narrowest bodied fish should be caught in experimental traps.**

*L350-362 Not very clear.*

**We have rewritten the paragraph to make it clearer and to incorporate the revised results.**

**Original text:**

“We found no significant effect of escape gaps on mean fish length, total fish biomass, number of fish, Shannon diversity index, and species richness per trap haul. Despite a lack of differences in these measures of catch, more detailed analysis of the data at the family and species level did reveal potential weak effects of escape gaps on catch: several thick bodied fish families were present in larger proportions in experimental traps compared to control traps, black durgon (a thin-bodied triggerfish) were absent in the experimental traps but formed 2.1% of all fish in control traps, and there was a greater proportion of small-sized (less than 15 cm length) fish in the control traps (though not significant). However, of the only two species with significantly different length-frequency distributions, one (lane snapper) had a larger mean length and a greater proportion of large fish in the control traps, contrary to expectations. Conversely the other species (rock beauty) had a larger mean length in the experimental traps, and the smaller size classes that were present in the control traps were absent in the experimental traps.”

**Rewritten (Lines 363 – 376):**

**“We found no significant effect of escape gaps on mean fish length, total fish biomass, number of fish, Shannon diversity index, and species richness per trap haul. Despite a lack of differences in these measures of catch, more detailed analysis of the data did reveal effects of escape gaps on catch. Specifically, the narrowest fish were found in significantly higher proportions in control traps compared to experimental traps and conversely the widest fish were found in higher proportions in experimental traps than control traps. Additionally, black durgon (a narrow-bodied triggerfish) were absent in the experimental traps but formed 2.1% of all fish in control traps and there was a greater proportion of small-sized (less than 15 cm length) fish in the control traps (though not significant). Furthermore, of the 9 species for which we had length at maturity values, 5 had a higher percentage of immature fish caught by the control trap compared to the experimental traps, with only 1 species having a higher percentage in the experimental trap, though these results were not statistically significant due to the relatively small magnitude of the differences. Taken together, these results suggest the escape gaps allowed some narrow bodied and immature fish to escape.”**

*L379-381 The escape through the mesh or entry funnel could happen in both experimental and control traps and if that occurs the gaps seem to be not working as wished to.*

**Agreed. This is why we list this as a potential reason we are not seeing a big difference between control and experimental traps (lines 404-406): “The relatively free movement of smaller sized fish in and out of all traps would explain the limited difference between experimental and control traps, but not the shift to larger sized fish with increasing soak time.”**

*L388-391 That means that the tested gaps are probably not necessary.*

**Whether escape gaps are a worthwhile investment is ultimately a decision made by fisheries managers. However, our results and previous experiments (see Table 1) show that escape gaps can have ecological and fisheries benefits by allowing immature fish to escape, albeit the magnitude of these benefits may be small.**

*L402-403 When you have a low and unbalanced N, and the result is not significant, it is better to not rely on the result.*

**Though the evidence for the difference in trap catches on leeward and windward sides of the island was weak (present in most parsimonious models for number of fish and species richness, but p = 0.08 and p = 0.11 respectively), we specifically state that this is a non-significant trend that fits our knowledge of the ecology of the islands. In this light, we do not think we are overstating the result, particularly since p < 0.05 is an arbitrary threshold and some use p < 0.1 for statistical significance.**

*L405-407 This is not entirely correct, because in this study there was a greater species richness in windward in spite of the lower N in this location…*

**There was actually lower species richness in windward locations, but there was an error in our results which stated the opposite (thanks to the reviewer for catching this!). This has now been corrected (lines 348-351):**

**“**Traps in windward locations have a trend of catching fewer fish (6.9, 95% CI 4.8 - 10.2) than leeward locations (9.9, 95% CI 8.3 - 11.8; Fig 6), ~~but~~ **and** also catch ~~more~~ **fewer** species of fish (**3.1 vs.** 4.0 ~~vs. 3.1~~ after 14 day soak time, 95% CIs: **2.4 - 4.1;** 3.5 - 4.5; ~~2.4 - 4.1;~~ Fig 6).**”**

*L409-412 This is a little bit, wishful thinking, because the results did not show a reduction in juvenile fish.*

**This sentence has been rephrased in light of the revised analysis suggested by the reviewer (lines 424 – 427): “The potential reduction in immature and narrow-bodied fish in the catch could have ecological and fisheries benefits, but given the relatively weak effect of the gaps, larger gaps may be more beneficial, though these should be trialled before being introduced.”**

**Our results do now show a reduction in immature (juvenile) fish in the experimental traps compared to the control traps. Furthermore, we are not suggesting that larger gaps should be used in management immediately, but rather that they should be trialled.**

*L412-414 There is no data to support that with soak time inferior to 4 days it would gave better results.*

**Lines 427- 429, changed text:** “Our results contrast with previous experiments, and indicate there may only be modest sustainability benefits of escape gaps at longer soak times ~~(4+ days)~~, though those longer soak times might naturally catch fewer small sized fish.”

**We provide a reasonable hypothesis, backed up by our data, why our longer soak times (though not specifically 4+ days) may see less difference in catch between control and experimental traps compared to past experiments. Given that all previous experiments with short (mostly 1 day) soak times showed an experimental effect, it is reasonable to believe that this would be the case here.**

**We state in the discussion lines (380 - 385): “**One possible explanation for the limited difference between our control and experimental trap catch is that the longer soak times of our traps resulted in most smaller fish exiting or being consumed in both trap types before hauling. The mean soak time for all traps was 11 days (range 4 - 30), which is much higher than all past experiments, which have mostly had 1 day soak times (Table 1)**”**

## References

[Fulton, S., López-Sagástegui, C., Weaver, A.H., Fitzmaurice-Cahluni, F., Galindo, C., Fernández-Rivera Melo, F., Yee, S., Ojeda-Villegas, M.B., Fuentes, D.A. & Torres-Bahena, E. (2019). Untapped Potential of Citizen Science in Mexican Small-Scale Fisheries. *Front. Mar. Sci.*, 6.](https://www.zotero.org/google-docs/?M82JzW)

[Oviedo, A.F.P. & Bursztyn, M. (2017). Community-based monitoring of small-scale fisheries with digital devices in Brazilian Amazon. *Fish. Manag. Ecol.*, 24, 320–329.](https://www.zotero.org/google-docs/?M82JzW)

[Roelfsema, C., Thurstan, R., Beger, M., Dudgeon, C., Loder, J., Kovacs, E., Gallo, M., Flower, J., Cabrera, K.-L.G., Ortiz, J., Lea, A. & Kleine, D. (2016). A citizen science approach: A detailed ecological assessment of subtropical reefs at point lookout, Australia. *PLoS ONE*, 11.](https://www.zotero.org/google-docs/?M82JzW)
